# Progression of atherosclerosis with carnitine supplementation: a randomized controlled trial in the metabolic syndrome

**DOI:** 10.1186/s12986-022-00661-9

**Published:** 2022-04-02

**Authors:** Amer M. Johri, Marie-France Hétu, Daren K. Heyland, Julia E. Herr, Jennifer Korol, Shawna Froese, Patrick A. Norman, Andrew G. Day, Murray F. Matangi, Erin D. Michos, Stephen A. LaHaye, Fraser W. Saunders, J. David Spence

**Affiliations:** 1 Department of Medicine, Cardiovascular Imaging Network at Queen’s University, Kingston, ON Canada; 2 Department of Critical Care Medicine, Clinical Evaluation Research Unit, Kingston, ON Canada; 3 Kingston Health Sciences Centre, Kingston, ON Canada; 4 The Kingston Heart Clinic, Kingston, ON Canada; 5 Division of Cardiology, Johns Hopkins University School of Medicine, Baltimore, USA; 6 Department of Medicine, Queen’s University, 76 Stuart Street, KGH FAPC 3, Kingston, ON K7L 2V7 Canada; 7 Southeastern Ontario Vascular Laboratory, Kingston Health Sciences Centre, Kingston, ON Canada; 8 Stroke Prevention and Atherosclerosis Research Centre, University of Western Ontario, London, ON Canada

**Keywords:** L-carnitine, Cardiovascular, Carotid, Plaque, Ultrasound

## Abstract

**Background:**

L-carnitine (L-C), a ubiquitous nutritional supplement, has been investigated as a potential therapy for cardiovascular disease, but its effects on human atherosclerosis are unknown. Clinical studies suggest improvement of some cardiovascular risk factors, whereas others show increased plasma levels of pro-atherogenic trimethylamine N-oxide. The primary aim was to determine whether L-C therapy led to progression or regression of carotid total plaque volume (TPV) in participants with metabolic syndrome (MetS).

**Methods:**

This was a phase 2, prospective, double blinded, randomized, placebo-controlled, two-center trial. MetS was defined as ≥ 3/5 cardiac risk factors: elevated waist circumference; elevated triglycerides; reduced HDL-cholesterol; elevated blood pressure; elevated glucose or HbA1c; or on treatment. Participants with a baseline TPV ≥ 50 mm^3^ were randomized to placebo or 2 g L-C daily for 6 months.

**Results:**

The primary outcome was the percent change in TPV over 6 months. In 157 participants (L-C N = 76, placebo N = 81), no difference in TPV change between arms was found. The L-C group had a greater increase in carotid atherosclerotic stenosis of 9.3% (*p* = 0.02) than the placebo group. There was a greater increase in total cholesterol and LDL-C levels in the L-C arm.

**Conclusions:**

Though total carotid plaque volume did not change in MetS participants taking L-C over 6-months, there was a concerning progression of carotid plaque stenosis. The potential harm of L-C in MetS and its association with pro-atherogenic metabolites raises concerns for its further use as a potential therapy and its widespread availability as a nutritional supplement.

*Trial registration*: ClinicalTrials.gov, NCT02117661, Registered April 21, 2014, https://clinicaltrials.gov/ct2/show/NCT02117661.

**Supplementary Information:**

The online version contains supplementary material available at 10.1186/s12986-022-00661-9.

## Background

Metabolic syndrome (MetS) is a group of cardiovascular risk factors that when combined increase the risk of heart disease and stroke. The risk factors include obesity, hypertension, high blood glucose, low high-density lipoprotein cholesterol (HDL-C), and high triglycerides [1]. Any combination of three risk factors determines predisposition to MetS. Individuals with MetS are three times more likely to have a heart attack or stroke due to atherosclerosis [2].

L-carnitine (L-C) is a non-essential dietary amino acid synthesized from lysine and methionine, involved in adenosine triphosphate and energy generation [3–5]. L-C is found to be naturally occurring in red meat, dairy, and poultry products [6]. Ubiquitous to the billion dollar global supplement industry, L-C usage is unregulated, and a widely available ingredient touted in energy drinks, pre-workout supplements, and a large proportion of other products found at health food stores [7, 8].

L-C and its short esters affect lipid metabolism by acting as obligatory cofactors for oxidation of fatty acids and facilitating the transport of long-chain fatty acids across the mitochondrial membrane. Oral administration of L-C has been shown to help in lipid metabolism [9, 10], insulin-dependent glucose disposal, and blood pressure control [11]; suggesting a potential favorable influence on the cardiovascular risk factors associated with MetS. However, there has been debate as to whether L-C can provide benefit to individuals with cardiovascular disease, and its effects remain unknown with no randomized controlled trials assessing direct effects on human atherosclerosis to date. Though epidemiological studies [12] and randomized trials [9] have suggested a possible reduction of cardiovascular risk factors following treatment, other animal and human studies have shown that L-C may increase pro-atherogenic metabolites such as trimethylamine oxide (TMAO) [13–15]. Thus, a randomized controlled trial directly assessing atherosclerosis in patients at risk of cardiovascular disease is required.

Three dimensional (3D) carotid ultrasound is a sensitive method of quantifying atherosclerotic plaque progression and is capable of directly detecting changes in atherosclerotic plaque progression (burden and degree of stenosis) within three months [16, 17]. The purpose of this study was to determine if L-C causes progression or regression of carotid plaque burden and occlusion in MetS participants, by directly quantifying the change in plaque volume and stenosis over six months of treatment.

## Methods

### Study design

This was an investigator-initiated phase 2, prospective, parallel, double blinded, randomized, placebo-controlled, two-center trial (ClinicalTrials.gov Identifier: NCT02117661, the ECoM trial). Eligible patients were randomized following a two-step inclusion process and randomized (first dose) within four weeks of initial carotid ultrasound. A centralized web-based randomization system was used to randomly allocate participants to study groups. Randomization was performed using permuted blocks of variable size and stratified by site.

### Trial participants

Patients, followed in clinics for cardiovascular disease prevention, fulfilling diagnostic criteria for MetS were recruited from two centres: The Stroke Prevention and Atherosclerosis Research Centre (SPARC) in London, Ontario, and the Cardiovascular Imaging Network at Queen’s (CINQ), Kingston, Ontario. Following randomization, participants entered a six-month treatment period with either 2 g/day of oral L-C or placebo (cellulose), both given in divided doses in the morning (2 × 500 mg) and evening (2 × 500 mg). To maintain blinding, the placebo was identical in appearance to the L-C capsule, but contained cellulose powder as the ingredient. Both capsules were prepared by the Commissioner’s Pharmacy, London, ON and Wynand Bekker Pharmacy Ltd.-Shoppers Drug Mart, Belleville, ON. All batches were tested for contaminants (Analytical Services Unit at Queen’s and Canadian Analytical Laboratories Inc).

MetS patients were assessed for inclusion and exclusion in two steps: **STEP 1:** Participants meeting initial eligibility screening were invited to participate, approached for informed consent, and provided blood sample (fasting) for confirmation of MetS. **STEP 2:** Participants received a 3D carotid ultrasound. A minimal total plaque volume (TPV) of ≥ 50 mm^3^ was required for eligibility. Participant meeting both step 1 (confirmation of MetS by blood and waist measurement) and step 2 (minimum plaque volume) were selected for randomization.

**Fig. 1:**
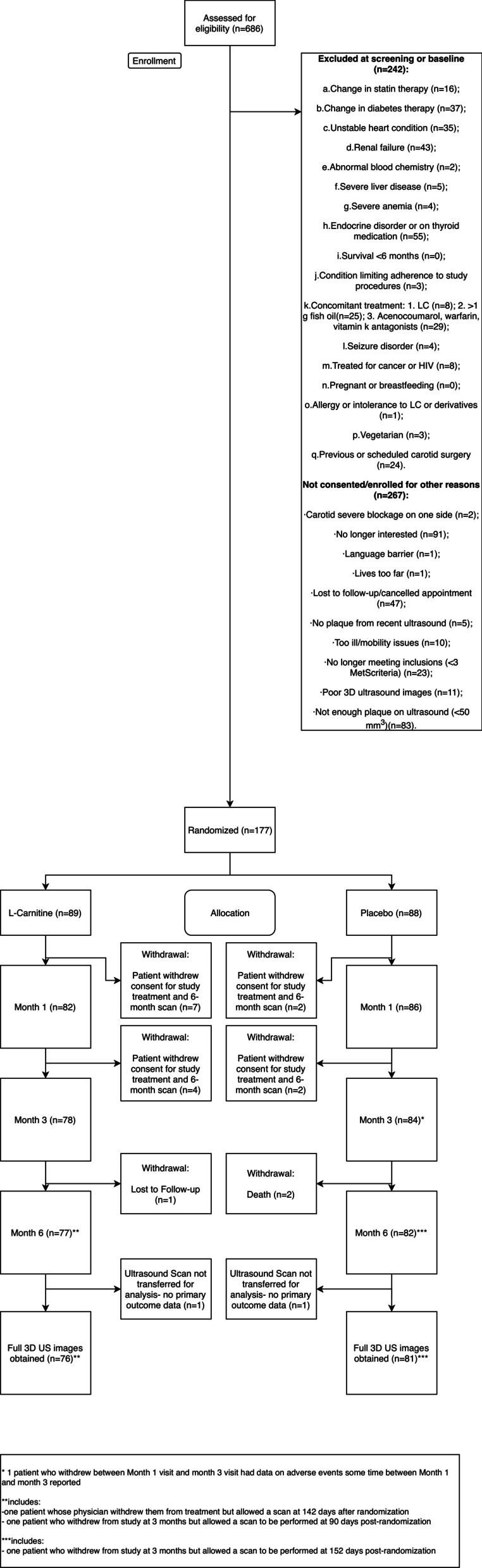
CONSORT participant flow diagram

Participants were randomized within 2 weeks of the 1st visit and given the first dose. Randomization codes and blinding were coordinated by the site’s designated pharmacist. A blinded study coordinator at each site enrolled and assigned participants to a kit number based on the web-based system output at the randomization visit. Research coordinators, participants, and investigators were blinded to allocation. The CONSORT diagram is presented in Fig. 1.

### Inclusion criteria

**STEP 1:** Men and women, > 18 years, meeting the criteria for clinical diagnosis of MetS, according to the International Diabetes Federation [16], where any 3 or more of the 5 following risk factors constituted a diagnosis of MetS:

- Elevated waist circumference: males > 102 cm and females > 88 cm.
- Elevated triglycerides: > 1.7 mmol/L (150 mg/dL).
- Reduced HDL-C: < 1.0 mmol/L (40 mg/dL) in males; < 1.3 mmol/L (50 mg/dL) in females or treated.
- Elevated BP: Systolic > 130 and/or diastolic > 85 mm Hg or treated.
- Elevated fasting glucose: > 5.6 mmol/L (100 mg/dL), or HbA1c ≥ 6.2%, or treated.

**STEP 2:** Carotid ultrasound confirmation of a baseline TPV ≥ 50 mm^3^ by 3D US, to ensure sufficiently detectable plaque.

### Exclusion criteria

1. A change in statin or diabetes therapy dosing in the previous three months;
2. Unstable or untreated arrhythmia, angina or heart attack; Symptomatic heart failure;
3. Severe liver disease, severe anemia, severe abnormal blood biochemistries, or renal failure (eGFR < 50 mL/min/1.73m^2^);
4. Endocrine disorders;
5. A condition expected to limit survival to less than 6-months;
6. A condition limiting adherence (i.e. alcoholism, drug addiction, known poor adherence);
7. Concomitant treatment with: anticonvulsants, L-C or derivatives,  Acenocoumarol, Warfarin, > 1 g fish oil, and/or drugs that affect insulin sensitivity, and/or thyroid treatment;
8. A seizure disorder or at risk of seizure; treatments for cancer or HIV infection (secondary L-C deficiency);
9. Currently pregnant or breastfeeding;
10. History of allergy to L-C or derivatives;
11. Vegetarianism due to potential for altered L-C metabolism;
12. Previous carotid surgery.

### Study visits

Following randomization, visits were at month 1, 3, and 6 (study exit). Carotid scans to assess plaque burden were conducted at baseline and study exit. Participants’ weight, waist circumference, and food (portions of red meat consumed per week) and exercise diaries were collected. An average of 3 diaries were used for analysis. Leftover pill counts were counted at each visit. Fasting blood sample and 3D ultrasound were taken at baseline and month 6 (or study exit if participant withdrew consent after the 3 month mark).

### Carotid ultrasound

A focussed carotid ultrasound limited to measuring carotid intimal media thickening (CIMT) and plaque measurements was conducted using a Philips Healthcare IU22 system equipped with a L12-3 linear transducer for 2D and a VL13-5 transducer for 3D acquisition (Philips Healthcare, Markham, ON, Canada). Identical equipment and methods were used at both sites. Images were acquired on the left and right sides of the neck. The 3D volume acquisition was performed using the 3D function, set at an angle of 15° to 30°. Resolution speed was turned down 1 notch counter clockwise and the harmonics turned off. Gain or focus was optimized for each participant. Images were stored in DICOM format and analysed offline by the core reading site (Kingston).

### Carotid variables measured

CIMT, maximum plaque height (MPH), total plaque area (TPA), total plaque volume (TPV), and maximum area stenosis caused by the plaque were quantified using QLAB version 10.2 (Philips Healthcare, Andover, MA, USA). CIMT was measured using the automatic function and averaged for both sides. Plaque height was measured manually in the bulb/internal carotid artery region using the caliper function, and the maximum height of either side was used in the analysis. Plaque area was traced manually and the total area of both sides added [18]. Plaque measurements were conducted as per the American Society of Echocardiography recommendations [19].

Plaque volume and lumen area reduction (stenosis) in 3D was quantified using QLAB VPQ. The acquisition video frames were analyzed in short-axis view of the bifurcation towards the internal carotid artery (ICA) in a series of image frames. The start (first frame) and end (last frame) of the plaque was outlined using an ellipse and set as “no plaque”, then a key frame in the center of the plaque was outlined to represent “plaque”. Reference key frames were manually modified as necessary. The TPV and maximum area reduction were automatically calculated. The sum of both right and left were added to give the TPV. A greater maximum area reduction (automatically calculated by the software) represented a smaller lumen to plaque proportion (increased area stenosis) [19].

### Statistics

Our sample size was based on the % difference of TPV from baseline to six months. Based on six month studies by Migrino et al. [20] and Tani et al. [21] measuring TPV, we estimated the standard deviation of the % change in TPV to be 11%. In a study by Ainsworth et al. [16] specifically designed to aid in estimating effect sizes for trials such as ours, it was estimated that after six months of treatment with atorvastatin, average TPV would regress by 180 mm^3^ from a baseline of 690 (i.e. a 26% reduction). We considered an absolute reduction of 5.2% in TPV (20% of 26% reduction observed with atorvastatin) to be both plausible and minimally clinically important. We required 72 completers per group to achieve 80% power at a 2-sided alpha = 0.05 to detect a 5.2% absolute difference in the % change between groups. We required 80 patients per group to allow for up to 10% lost to follow up.

The primary outcome of this study was the percent change in the TPV between the baseline visit and the study exit, 6 months later. Absolute changes are also reported. Secondary analyses of changes in additional plaque parameters were also assessed. A secondary planned outcome assessment of changes in LDL density, registered on clinicaltrials.gov, was not assessed due to the underdeveloped experimental nature of the assay. Analysis of covariance (ANCOVA) with treatment assignment as an indicator variable blocking by site and controlling for baseline plaque measures as a covariate was used to estimate the within-arm change and between-arm difference in change. The raw plaque measures were modeled to estimate the absolute change. Percent change (the primary outcome) within and between treatment arms were estimated by modelling the log of the ratio of the month 6 and baseline plaque measures and then exponentiating the relevant model parameter estimates, subtracting one, and multiplying by 100. Changes in clinical and biochemical characteristics were analyzed using the Wilcoxon rank-sum test. False Discovery Rates (FDR) were used to address multiplicity of hypothesis testing. Compliance was defined as the percentage of the study medication taken, based on returned capsules.

Subgroup analyses were performed for TPV and stenosis to assess potential effect modification of: baseline plaque levels (higher or lower than median), average weekly red meat consumption (higher or lower than median), sex (male or female), and study site. This analysis used the same ANCOVA model described above except we added an interaction term between treatment assignment and the given subgroup to estimate within-subgroup treatment effects and obtain *p* values testing for a difference in treatment effect between subgroups. We did not control for baseline TPV or stenosis when those variables defined the subgroups.

All analyses were performed using SAS Version 9.4 (SAS Institute Inc., Cary, NC, USA). A two-sided *p* value < 0.05 was considered statistically significant.

## Results

### Participant workflow

We studied the effects of L-C on carotid atherosclerosis in participants with MetS. The first patient was screened for inclusion on January 28, 2015. Between February 18, 2015 and January 6, 2017, 89 participants were randomized to L-C treatment, while 88 were randomized to a placebo (N = 177) [Fig. 1]. Follow-up was 6-months from randomization (end July 7, 2017). Of these, 76 and 81 participants from the L-C and placebo arms respectively, had complete carotid scan data and were included in the primary analysis (N = 157).

### Participant characteristics

Both participant arms were well balanced on all characteristics, though the intervention arm contained a slightly greater proportion of females than the placebo arm (33.7% female vs 25.0% female, respectively; Table 1). The two arms were reasonably well balanced with respect to the presence of cardiovascular comorbidities (angina, valvular disease, congestive heart failure, hypertension, diabetes, hyperlipidemia, and cardiac revascularization), with the placebo arm having marginally higher rates of cardiac arrhythmia (19% vs 11%), myocardial infarction (MI) (39% vs 30%), and obesity (BMI ≥ 30 kg/m^2^ 78% vs 66%). In terms of concomitant cardiac medications, the two arms were well balanced (statins [95% of participants], angiotensin converting enzyme inhibitors, angiotensin II receptor blockers, anti-platelet, anti-coagulation, diabetes medications, calcium channel blockers, diuretics, anti-anginal, and nonsteroidal anti-inflammatory drugs—NSAIDs), though the placebo arm had more participants on beta-blockers (49% vs 33%) and fewer participants on alpha-blockers (7% vs 12%).

**Table 1:** Baseline clinical and blood characteristics

Variable	L-C (n = 89)	Placebo (n = 88)	Total Cohort (n = 177)
Age (years), mean ± SD	66.5 ± 8.2	66.3 ± 8.3	66.4 ± 8.2
Sex (male), N (%)	59 (66.3)	65 (73.9)	124 (70.1)
Race/ethnicity (Caucasian), N (%)	87 (97.8)	87 (98.9)	174 (98.3)
WC (cm), mean ± SD	111.6 ± 12.8	112.8 ± 12.0	112.2 ± 12.4
Waist: Hip ratio, mean ± SD	1.0 ± 0.1	1.0 ± 0.1	1.0 ± 0.1
BMI (kg/m^2^), mean ± SD	32.2 ± 5.7	32.5 ± 5.2	32.4 ± 5.4
Current smoker, N (%)	12 (13.5)	8 (9.1)	20 (11.3)
HDL-C (mmol/L), median [IQR]	1.1 [0.9 to 1.3]	1.1 [1.0 to 1.3]	1.1 [1.0 to 1.3]
Triglycerides (mmol/L), median [IQR]	1.3 [0.9 to 1.8]	1.4 [0.9 to 1.9]	1.4 [0.9 to 1.9]
Glucose (mmol/L), median [IQR]	6.0 [5.2 to 7.2]	5.9 [5.2 to 6.7]	6.0 [5.2 to 7.0]
HbA1C (%), median [IQR]	6.2 [5.9 to 7.3]	6.5 [5.8 to 7.0]	6.4 [5.9 to 7.1]
eGFR (mL/min/1.73 m^2^), mean ± SD	83.3 ± 17.0	80.7 ± 17.0	82.0 ± 17.0
Systolic BP (mmHg), mean ± SD	130.5 ± 18.1	132.6 ± 18.8	131.5 ± 18.4
Diastolic BP (mmHg), mean ± SD	76.1 ± 10.1	78.1 ± 10.5	77.1 ± 10.4
Total of MetS criteria			
3, N (%)	24 (27.0)	20 (22.7)	44 (24.9)
4, N (%)	45 (50.6)	47 (53.4)	92 (52.0)
5, N (%)	20 (22.5)	21 (23.9)	41 (23.2)
Weekly consumed servings of red meat, mean ± SD	2.9 ± 2.9	2.8 ± 2.3	2.8 ± 2.6

*p* values are not presented due to the nature of randomization. For HDL-C, to convert from mmol/L to mg/dL, multiply by 38.67. For triglycerides, to convert from mmol/L to mg/dL, multiply by 88.57

BMI, body mass index; BP, blood pressure; eGFR, estimated glomerular filtration rate; HbA1C, hemoglobin A1c; HDL-C, high-density lipoprotein cholesterol; L-C, L-carnitine; MetS, metabolic syndrome, WC, waist circumference

### Compliance

Both arms had similarly high compliance to the study treatment, with the average compliance across all 6 months being 95.4% and 95.7% in the intervention and placebo arms, respectively. Above 80% compliance rates were within 2% of each other for all months; with intervention ranging from 92.0% to 96.0%, and the placebo ranging from 93.9% to 97.6%.

### Safety

Known L-C adverse effects recorded included: gastrointestinal symptoms, a fishy body odour, and occurrence of a seizure. Adverse events collected were: angina (change in frequency), stroke, transient ischemic attack, MI, congestive heart failure, cardiac revascularization, cerebral revascularization, peripheral revascularization, high blood pressure and diabetes (new diagnosis only). No important differences between arms in adverse effects or adverse events were observed at any time point. No seizure occurred.

Serious adverse events (SAEs) were collected at each visit and reported according to institutional policies. A total of seven participants had a serious adverse event (four in the intervention arm, three in the placebo arm). Only one SAE was deemed “unlikely related” to the intervention, with the rest deemed “not related”. The SAE deemed “unlikely related” was in the placebo arm.

### Outcomes

**Fig. 2:**
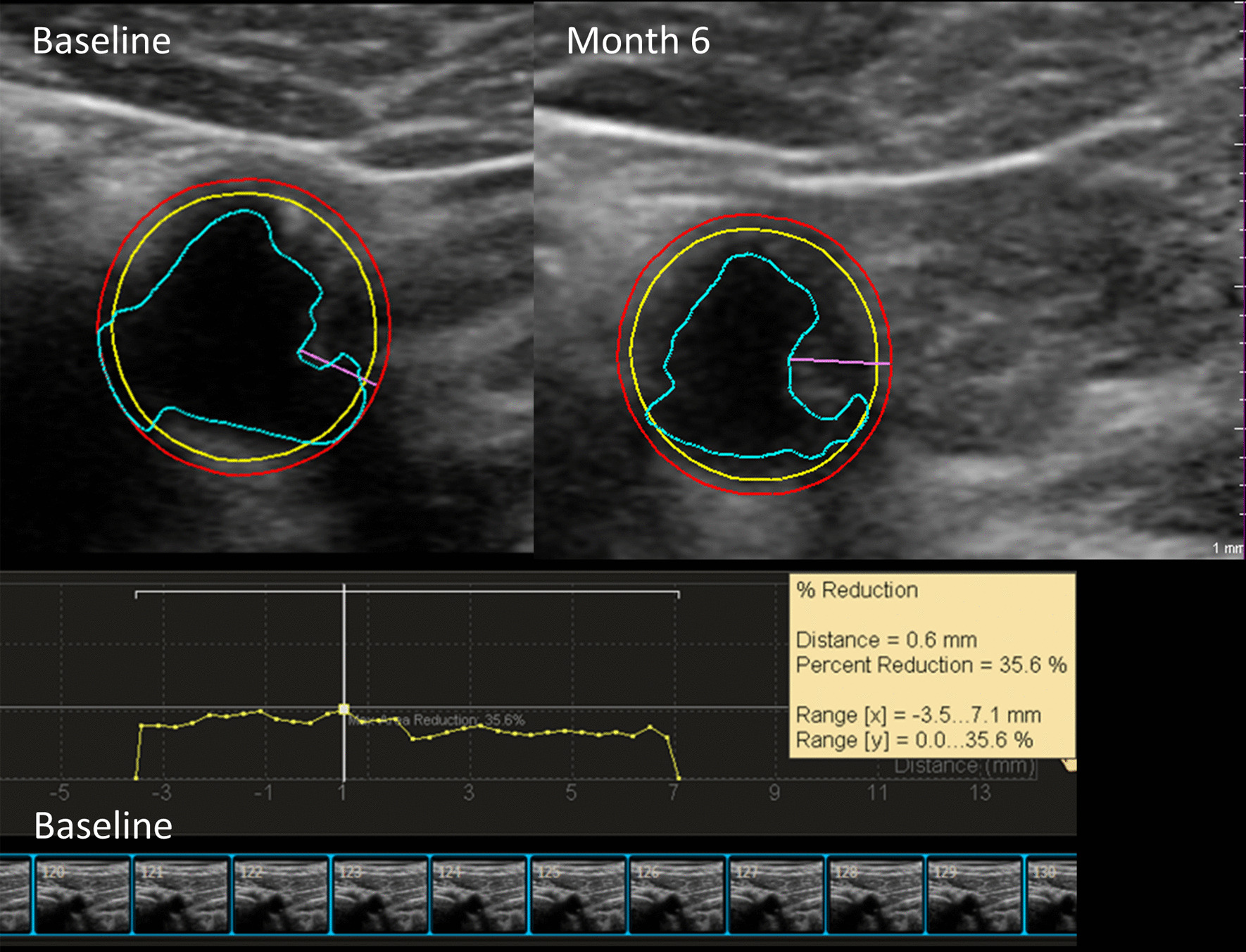
Quantification of carotid arterial plaque acquired by three-dimensional ultrasound. Philips VPQ software was used to assess plaque in 3D. A cross section of the vessel demonstrates a percent lumen reduction (stenosis) of 36% at baseline compared to 47% at month 6

Baseline plaque burden was well balanced between groups (Additional file 1: Table S1). The changes in plaque variables were calculated as the absolute and percent change from baseline to month 6 (Table 2). Figure 2 illustrates an example of the quantification method for TPV and stenosis. No statistically significant difference between arms was found for either absolute or percent TPV change. However, there was a significant between group difference for an increase in carotid arterial plaque stenosis (significant progression). Specifically, the % change of in the L-C intervention arm was 9.3% (p = 0.02) greater than in the placebo arm, which corresponded to an absolute between arm difference of 2.7% (*p* = 0.03).

**Table 2:** Primary outcome total plaque volume percent change and absolute change from baseline to month 6 and exploratory plaque variables

Variable	L-C Mean change (95%CI)	L-C *p* value	Placebo Mean Change (95%CI)	Placebo * p* value	Between-Arm Diff. Mean (95%CI)	Between-Arm Diff *p* value
Percent change						
TPV (%)	− 2.4 (− 5.3 to 0.6)	0.12	− 1.2 (− 4.0 to 1.8)	0.44	− 1.2 (− 5.3 to 3.0)	0.56
Stenosis (%)	4.6 (− 0.7 to 10.3)	0.09	− 4.3 (− 9.0 to 0.7)	0.09	9.3 (1.6 to 17.6)	0.02*
CIMT (%)	0.70 (− 1.8 to 3.3)	0.58	0.40 (− 2.0 to 2.9)	0.75	0.30 (− 3.2 to 3.9)	0.86
MPH (%)	0.1 (− 3.5 to 3.9)	0.95	2.8 (− 0.9 to 6.6)	0.14	− 2.6 (− 7.6 to 2.6)	0.32
TPA (%)	− 1.7 (− 6.7 to 3.6)	0.52	− 1.5 (− 6.3 to 3.6)	0.57	− 0.2 (− 7.3 to 7.4)	0.95
Absolute change						
TPV (mm^3^)	− 7.5 (− 18.9 to 4.0)	0.20	0.5 (− 10.6 to 11.6)	0.93	− 8.0 (− 24.0 to 8.0)	0.33
Stenosis (%)	1.6 (− 0.2 to 3.4)	0.07	− 1.1 (− 2.8 to 0.7)	0.22	2.7 (0.2 to 5.2)	0.03*
CIMT (mm)	0.0 (− 0.0 to 0.0)	0.58	0.0 (− 0.0 to 0.0)	0.43	− 0.0 (− 0.0 to 0.0)	0.88
MPH (mm)	0.0 (− 0.1 to 0.1)	0.79	0.1 (0.0 to 0.3)	0.046*	− 0.1 (− 0.3 to 0.1)	0.23
TPA (mm^2^)	− 0.7 (− 3.3 to 1.9)	0.60	− 0.3 (− 2.8 to 2.3)	0.83	− 0.4 (− 4.1 to 3.3)	0.82

Statistical test: analysis of covariance controlling for site and baseline

CIMT, carotid intima-media thickness; L-C, L-carnitine; MPH, maximum plaque height; TPA, total plaque area; TPV, total plaque volume

*Significant

Clinical characteristics and biochemical values from baseline and month 6 were compared and the change in median calculated (Additional file 1: Table S2). From baseline to 6 months, the two arms were found to be significantly different with regard to change in total carnitine, free carnitine, magnesium, total cholesterol, LDL-C, waist: hip ratio, systolic blood pressure, diastolic blood pressure, and meat consumption. However, only total carnitine, free carnitine (due to L-C supplementation), and magnesium change maintained a false discovery rate (FDR) of < 0.05 after adjusting for multiplicity of outcomes.

**Fig. 3:**
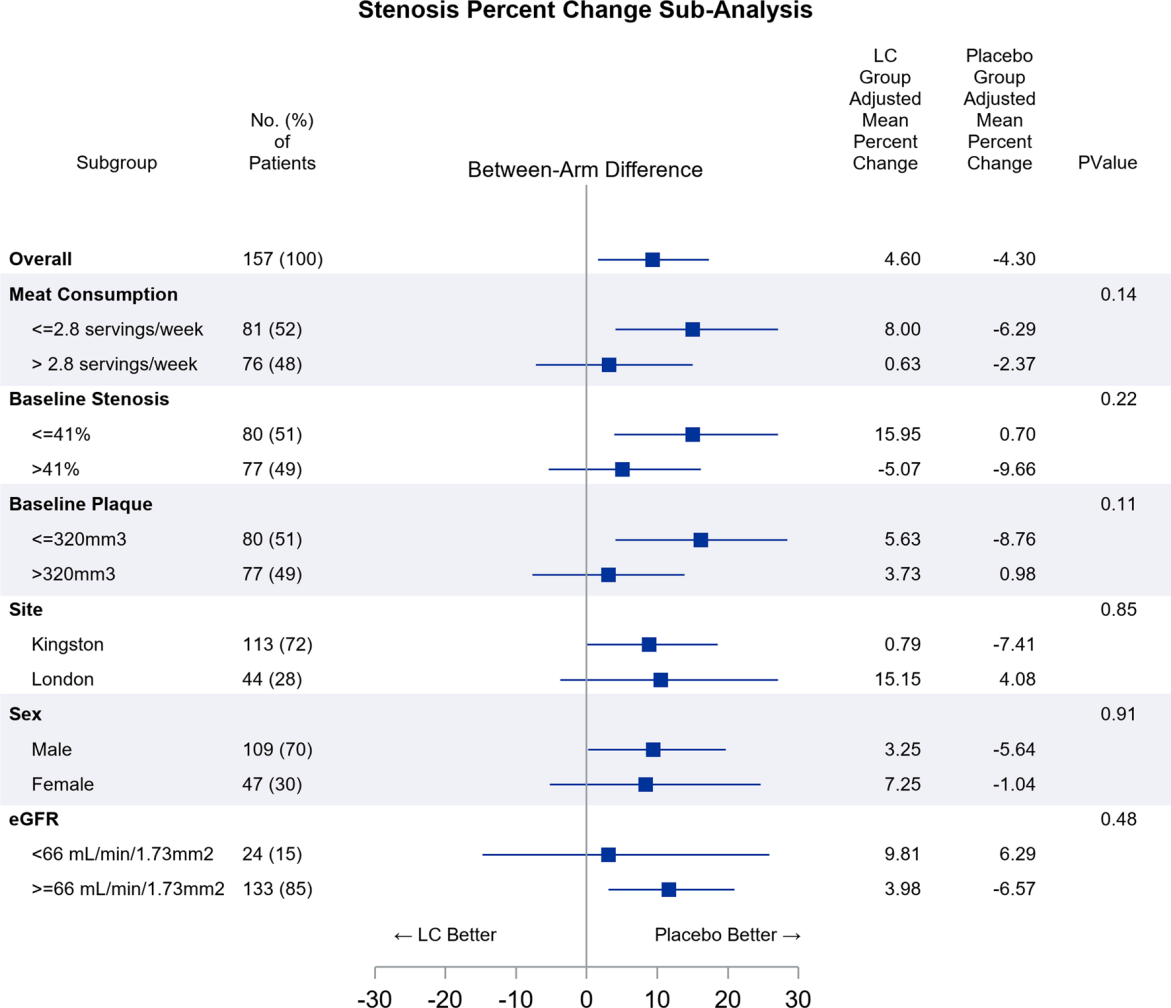
Forest plot of stratified sub-analysis of percent change in stenosis. Although we found significant differences in percent increase of carotid stenosis within sub-groups, no tests for treatment arm by sub-group interactions reached *p* < 0.05. LC, L-carnitine. N = 157

We performed subgroup analyses to determine if the treatment effect of L-C on TPV or maximum area stenosis differed by red meat consumption, baseline stenosis, baseline TPV, recruitment site, sex, or eGFR. There were no significant between-arm differences in TPV for any subgroups (data not shown). Compared to the placebo arm, the L-C arm had a significantly greater percent increase in stenosis among patients who ate less red meat (percent difference 15.2%; *p* = 0.01), had lower baseline stenosis (15.2%, *p* = 0.01), had lower baseline TPV (15.8%; *p* = 0.01), were males (9.4%; *p* = 0.046) and had high eGFR (11.3%; *p* = 0.01), while there were no significant between arm differences in the complement subgroups. However, no tests for treatment arm by sub-group interactions were statistically significant (Additional file 1: Table S3 and Fig. 3).

## Discussion

This randomized controlled trial is the first to directly demonstrate a measurable progression in the degree atherosclerotic carotid plaque stenosis in response to carnitine supplementation over a 6-month period. No previous studies have assessed the direct effects of L-C therapy on atherosclerotic plaque, as conducted here; however, other recent work has indirectly suggested an unfavourable association between L-C and cardiovascular risk factors [22, 23]. Given the widespread availability of carnitine and its unregulated usage in the general population, this carefully conducted clinical trial has significant implications for cardiovascular risk of populations.

Previous studies, though not RCT, have also raised concerns with respect to L-C and association with cardiovascular risk factors. Gao et al. [22] recently conducted a cross-sectional study in 1,081 participants in Newfoundland, Canada to determine correlations between serum carnitine levels and cardiovascular markers. Though not all participants met the criteria for MetS, serum carnitine levels (not taking L-C supplements) were associated with components of MetS: serum triglycerides (r = 0.13) and serum insulin (r = 0.12) in males with normal fasting glucose and with serum triglycerides (r = 0.14) in hyperglycemic males. In females, positive correlations were identified between serum L-C and obesity, total cholesterol, glucose, insulin, and insulin resistance in those with normal fasting glucose level, but not if hyperglycemic. Similarly, our placebo-controlled trial confirms an increase in total cholesterol and LDL-C levels in participants taking L-C, suggesting a potential mechanism linking L-C to the risk of increased atherosclerosis.

The pro-atherogenic effects of L-C therapy observed by our work and others, may be mediated through an increased production of TMAO [13]. Samulak et al. [24] showed greater TMAO production in women randomized to 1500 mg L-C for 6 months, compared to placebo (N = 18). Neither lipid profiles nor inflammatory markers were modified by either the carnitine supplementation or the washout period. TMAO levels returned to normal within 4 months of supplement cessation. As these were healthy women, our findings suggest a different effect or mechanism in individuals with MetS.

The pro-atherogenic effects of L-C and TMAO are relevant to the recommendations by the American Heart Association and other Societies to decrease red meat consumption in favour of other lean protein sources [25]. The carnitine-TMAO pathway has been suggested as a possible explanation for increased atherosclerotic risk in red meat eaters [6, 13]. Wang et al. [23] demonstrated that switching from red meat consumption to white meat or non-meat protein reduced plasma and urine TMAO over one month. Red meat ingestion reduced fractional renal excretion of TMAO, but increased fractional renal excretion of carnitine and two additional gut microbiota-generated metabolites of carnitine: c-butyrobetaine, and crotonobetaine. Red meat or white meat (vs. non-meat) increased TMA and TMAO production from carnitine, but not choline. Interestingly, we observed an increase in the degree of atherosclerotic carotid stenosis in the L-C group who consumed less red meat (< 2.8 servings per week, 15.2% increase) compared to placebo, but not in the high meat-eating group. However, the test for interaction did not reach p < 0.05. Nevertheless, it may be that low meat eaters are less adapted to processing carnitine intake than high meat eaters. The mechanism associated with L-C may have been amplified in participants who do not habitually eat large amounts of red meat, as the overall change in TMAO levels may have been greater suggesting a future avenue of further study.

Though our study indicated an adverse effect of L-C on carotid stenosis (degree of atherosclerotic occlusion), the overall plaque volumes measured did not progress, likely because plaque burden and stenosis are different phenotypes [26]. Due to compensatory enlargement (Glagov’s phenomenon) [27], arteries enlarge with progression of plaque, but they do not necessarily narrow. Stenosis and occlusion are most likely the consequence of plaque rupture and thrombosis. This is illustrated by a study reporting that Lp(a) was associated with thrombosis [28], stenosis, and occlusion, but not plaque burden [29]. In multiple regression models, Lp(a) was found to be a significant independent predictor of stenosis (*p* < 0.001) and vessel occlusion (*p* = 0.02), but not plaque area [29]. An increase in stenosis but not plaque volume may be explained by effects of TMAO on arterial thrombosis [30–33]. In our study, even though plaque stenosis was not a pre-specified outcome, the finding remains concerning and important for potential harm.

We found a greater increase in stenosis in both arms in the London site participants, compared to the Kingston site. The London site had a higher proportion of participants with a history of stroke/TIA (55% vs 11%, *p* < 0.001), while the Kingston site had a higher proportion of participants with a history of MI (41% vs 11%, *p* = 0.001). TMAO is believed to enhance platelet hyper-reactivity and increase thrombosis risk. TMAO may be associated with changes in plaque composition or shape.

The supplement industry is estimated to be worth nearly $300 billion globally, and nearly half of U.S adults report taking at least one vitamin or supplement [34], many doing so without any specific clinician recommendation. Individuals often consume L-C for boosting energy or build muscle strength. However, although this supplement is easily available without a prescription in energy drinks, health food stores, or as a protein supplement, this does not mean that it is necessarily benign or free from adverse effects. While secondary outcomes should be interpreted cautiously in the face of overall non-significant results for the primary outcome, our findings suggest potential harm with L-C supplements, both for worsening cardiometabolic risk factors (i.e., LDL-C) and for atherosclerosis progression (i.e., stenosis). This raises concern about the use of these supplements, particularly in patients at high cardiovascular risk such as those with the MetS.

### Limitations

The sample size and duration of studies may appear small, but when measuring TPV compared to CIMT our sample size was appropriately powered for the primary outcome [35]. Based on our previous study of effect of atorvastatin on TPV [16], our sample size was calculated to have sufficient power to show an effect size of (5%) within 6 months. However, the study was not powered to formally test effect modification between sub-group. Although all participants in this study had MetS, their underlying cardiovascular disease history may have different mechanisms and associations with stroke vs MI, requiring further investigation. Finally, though carotid plaque assessment by ultrasound is a surrogate marker, it has now been validated as a meaningful predictor of cardiovascular events, with robust recommendations on the performance, standardization and clinical utility in the prediction of important atherosclerotic outcomes [19].

## Conclusions

In this randomized, double-blinded, placebo-controlled trial of adults with the MetS, we did not observe a change in total plaque volume among participants treated with L-C for 6 months. However, secondary analyses indicated a greater progression of carotid stenosis with L-C supplementation compared to placebo. The adverse effect of L-C was greater in participants with low meat consumption than in those with high meat consumption. These findings raise concerns with respect to use of L-C as a therapy or supplement in individuals with MetS.

## Supplementary Information

**Additional file 1: Table S1.** Carotid plaque variables at baseline and month 6 between arms. **Table S2.** Change in clinical and blood characteristics from baseline to month 6. **Table S3**. Stratified sub-analysis of area stenosis percent change between arms.

## Data Availability

The datasets generated during and/or analysed during the current study are not publicly available due to patient privacy but are available from the corresponding author on reasonable request.

## Abbreviations

BP: Blood pressure
CI: Confidence interval
CIMT: Carotid intima-media thickness
eGFR: Estimated glomerular filtration rate
HbA1C: Hemoglobin A1c
HDL: High-density lipoprotein cholesterol
ICA: Internal carotid artery
L-C: L-carnitine
LDL-C: Low-density lipoprotein cholesterol
Lp(a): Lipoprotein (a)
MetS: Metabolic syndrome
MI: Myocardial infarction
MPH: Maximum plaque height
NSAIDs: Nonsteroidal anti-inflammatory drugs
SAEs: Serious adverse events
TMAO: Metabolites such as trimethylamine oxidase
TPA: Total plaque area
TPV: Total plaque volume
WC: Waist circumference
