# Supplementary material for: Progression of atherosclerosis with carnitine supplementation: a randomized controlled trial in the metabolic syndrome
Source: Nutr Metab (Lond). 2022 Apr 2;19:26. doi: 10.1186/s12986-022-00661-9 (PMC8976995; doi:10.1186/s12986-022-00661-9)
Supplement: Supplementary file 1 — Additional file 1: Table S1. Carotid plaque variables at baseline and month 6 between arms. Table S2. Change in clinical and blood characteristics from baseline to month 6. Table S3. Stratified sub-analysis of area stenosis percent change between arms. [file 12986_2022_661_MOESM1_ESM.docx]

**Table S1.** Carotid plaque variables at baseline and month 6 between arms.

| **Carotid Variable** | **Description** | **L-C** | **Placebo** | **Total Cohort** |
| --- | --- | --- | --- | --- |
| TPV Baseline (mm^3^) | Mean ±SD  median  [Q1 to Q3] | 425.4 ±316.8  329.0  [211.0 to 552.0] | 371.5 ±227.5 290.0  [216.0 to 494.0] | 397.6 ±274.8 320.0  [211.0 to 530.0] |
| TPV M6 (mm^3^) | mean ±SD  median  [Q1 to Q3] | 415.3 ±313.8 316.5  [215.0 to 487.0] | 369.7 ±240.0 307.0  [201.0 to 477.0] | 391.8 ±278.2 312.0  [204.0 to 485.0] |
| Stenosis Baseline (%) | mean ±SD median  [Q1 to Q3] | 41.0 ±15.4 43.0  [27.0 to 52.0] | 40.1 ±13.9 41.0  [29.0 to 49.0] | 40.5 ±14.6 41.0  [28.0 to 50.0] |
| Stenosis M6 (%) | mean ±SD median  [Q1 to Q3] | 42.4 ±15.6 41.0  [31.5 to 52.0] | 39.2 ±15.2 40.0  [26.0 to 51.0] | 40.7 ±15.4 41.0  [29.0 to 52.0] |
| CIMT Baseline (mm) | mean ±SD median  [Q1 to Q3] | 0.7 ±0.2 0.7  [0.6 to 0.8] | 0.7 ±0.1 0.7  [0.6 to 0.8] | 0.7 ±0.2 0.7  [0.6 to 0.8] |
| CIMT M6 (mm) | mean ±SD median  [Q1 to Q3] | 0.7 ±0.2 0.7  [0.6 to 0.8] | 0.7 ±0.2 0.7  [0.6 to 0.8] | 0.7 ±0.2 0.7  [0.6 to 0.8] |
| MPH Baseline (mm) | mean ±SD median  [Q1 to Q3] | 3.8 ±3.4 3.2  [2.6 to 3.9] | 3.1 ±1.0 3.0  [2.4 to 3.8] | 3.4 ±2.5 3.2  [2.5 to 3.9] |
| MPH M6 (mm) | mean ±SD median  [Q1 to Q3] | 3.8 ±3.9 3.2  [2.6 to 3.7] | 3.2 ±1.1 3.1  [2.5 to 3.9] | 3.5 ±2.8 3.2  [2.5 to 3.8] |
| TPA Baseline (mm^2^) | mean ±SD median  [Q1 to Q3] | 69.4 ±41.4 56.9  [42.9 to 93.1] | 57.1 ±34.6 49.0  [30.9 to 78.3] | 63.1 ±38.4 53.8  [35.4 to 83.1] |
| TPA M6 (mm^2^) | mean ±SD median  [Q1 to Q3] | 66.5 ±38.8 59.2  [37.8 to 85.8] | 56.9 ±35.6 48.7  [31.2 to 79.1] | 61.6 ±37.4 52.8  [33.2 to 84.0] |

CIMT, carotid intima-media thickness; L-C, L-carnitine; M6, month 6; MPH, maximum plaque height; TPA, total plaque area; TPV, total plaque volume. Note: p-values are not presented due to the nature of randomization.

**Table S2.** Change in clinical and blood characteristics from baseline to month 6.

| **Variable** | **L-C Median (Q1 to Q3)** | **Placebo Median (Q1 to Q3)** | **P-Value** | **FDR** |  |
| --- | --- | --- | --- | --- | --- |
| **Clinical** |  |  |  |  |  |
| Body Mass Index | 0.3 (-0.4 to 0.8) | 0.3 (-0.5 to 0.9) | 0.61 | 0.85 |  |
| Waist:Hip ratio | 0.0 (0.0 to 0.1) | 0.0 (0.0 to 0.0) | 0.02 | 0.08 | * |
| Heart rate | 0.0 (-2.0 to 4.0) | -0.5 (-8.0 to 4.0) | 0.24 | 0.57 |  |
| Systolic BP | -2.0 (-11.0 to 9.0) | -7.0 (-14.0 to 2.0) | 0.04 | 0.16 | * |
| Diastolic BP | -1.0 (-6.5 to 4.0) | -3.5 (-10.0 to 1.0) | 0.04 | 0.16 | * |
| Meat Consumption | 0.0 (-1.0 to 1.3) | 1.0 (0.0 to 2.0) | 0.02 | 0.08 | * |
| **Blood** |  |  |  |  |  |
| Total Carnitine | 27.5 (16.1 to 39.2) | -1.10 (-4.5 to 2.6) | <.001 | 0.02 | ** |
| Free Carnitine | 19.8 (13.0 to 30.9) | -0.30 (-4.3 to 2.8) | <.001 | 0.02 | ** |
| Magnesium | 0.0 (-0.0 to 0.0) | -0.01 (-0.0 to 0.0) | 0.003 | 0.04 | ** |
| Phosphorous | 0.0 (-0.1 to 0.1) | 0.0 (-0.1 to 0.0) | 0.89 | 0.91 |  |
| Potassium | 0.0 (-0.2 to 0.2) | 0.0 (-0.1 to 0.2) | 0.47 | 0.80 |  |
| eGFR | -1.0 (-5.5 to 3.0) | 0.0 (-5.0 to 4.0) | 0.37 | 0.77 |  |
| HDL-C | -0.04 (-0.1 to 0.1) | -0.05 (-0.1 to 0.0) | 0.78 | 0.89 |  |
| HbA1c | 0.0 (-0.2 to 0.1) | 0.0 (-0.2 to 0.2) | 0.57 | 0.85 |  |
| Total Cholesterol | 0.10 (-0.1 to 0.3) | -0.06 (-0.4 to 0.1) | 0.008 | 0.06 | * |
| LDL-C | 0.05 (-0.1 to 0.3) | -0.07 (-0.3 to 0.2) | 0.007 | 0.06 | * |
| Triglycerides | 0.10 (-0.2 to 0.4) | 0.11 (-0.2 to 0.4) | 0.73 | 0.89 |  |
| Glucose | -0.2 (-0.9 to 0.6) | 0.05 (-0.6 to 0.6) | 0.22 | 0.55 |  |
| Insulin | 6.5 (-16.5 to 27.0) | 4.0 (-25.0 to 28.0) | 0.49 | 0.80 |  |

*P-value <.05, ** both P-value and FDR <.05. eGFR, estimated glomerular filtration rate; HbA1C, hemoglobin A1c; HDL, high-density lipoprotein cholesterol; L-C, L-carnitine; LDL, low-density lipoprotein cholesterol; METS units, metabolic equivalent exercise units. Statistical test: Wilcoxon rank-sum test. False Discovery Rates (FDR) were used to address multiplicity of hypothesis testing.

**Table S3**. Stratified sub-analysis of area stenosis percent change between arms.

| **Stratification Factor**  **Cut-off** | **L-C**  **Mean Change (95%CI)** | **L-C**  **P-Value** | **Placebo**  **Mean Change (95%CI)** | **Placebo**  **P-Value** | **Between-Arm Diff. Mean (95%CI)** | **Between-Arm Diff.**  **P-Value** |
| --- | --- | --- | --- | --- | --- | --- |
| Weekly servings of red meat (interaction p=0.14) | | | | | | |
| ≤ 2.8 | 8.0% (0.6% to 15.9%) | 0.03* | -6.3% (-13.0% to 0.9%) | 0.09 | 15.2% (4.1% to 27.6%) | 0.01* |
| > 2.8 | 0.6% (-7.0% to 8.9%) | 0.87 | -2.4% (-9.2% to 4.9%) | 0.50 | 3.1% (-7.2% to 14.6%) | 0.57 |
| Baseline Stenosis (interaction p=0.22) | |  |  |  |  |  |
| ≤41 % | 16.0% (7.4% to 25.2%) | <.001* | 0.7% (-6.4% to 8.3%) | 0.85 | 15.2% (3.9% to 27.6%) | 0.01* |
| >41 % | -5.1% (-12.0% to 2.3%) | 0.17 | -9.7% (-16.4% to -2.4%) | 0.01* | 5.0% (-5.4% to 16.6%) | 0.35 |
| Baseline TPV (interaction p=0.11) | |  |  |  |  |  |
| ≤320 mm^3^ | 5.6% (-2.4% to 14.3%) | 0.17 | -8.8% (-15.2% to -1.8%) | 0.02* | 15.8% (4.5% to 28.2%) | 0.01* |
| >320 mm^3^ | 3.7% (-4.0% to 12.0%) | 0.35 | 1.0% (-6.6% to 9.2%) | 0.81 | 2.7% (-7.4% to 13.9%) | 0.61 |
| Site (interaction p=0.85) | | | | | | |
| Kingston | 0.8% (-5.4% to 7.4%) | 0.81 | -7.4% (-12.9% to -1.6%) | 0.01* | 8.8% (-0.2% to 18.7%) | 0.05† |
| London | 15.2% (3.9% to 27.7%) | 0.01* | 4.1% (-6.0% to 15.4%) | 0.44 | 10.6% (-3.7% to 27.0%) | 0.15 |
| Sex (interaction p=0.91) | | | | | | |
| Male | 3.3% (-3.2% to 10.2%) | 0.32 | -5.6% (-11.1% to 0.3%) | 0.06 | 9.4% (0.2% to 19.5%) | 0.046* |
| Female | 7.3% (-2.3% to 17.7%) | 0.14 | -1.0% (-10.3% to 9.2%) | 0.83 | 8.4% (-5.3% to 24.1%) | 0.24 |
| eGFR (interaction p=0.48) | | | | | | |
| ≥66 mL/min/1.73 m^2^ | 4.0% (-1.7% to 9.9%) | 0.17 | -6.6% (-11.6% to -1.2%) | 0.02* | 11.3% (2.8% to 20.4%) | 0.01* |
| <66 mL/min/1.73 m^2^ | 9.8% (-5.8% to 28.0%) | 0.23 | 6.3% (-5.5% to 19.5%) | 0.31 | 3.3% (-14.8% to 25.3%) | 0.74 |

*Significant; †Borderline significance; CI, confidence interval; L-C, L-carnitine; TPV, total plaque volume.
